# Biomechanical analysis of miniscrew-assisted molar distalization with clear aligners: a three-dimensional finite element study

**DOI:** 10.1093/ejo/cjad077

**Published:** 2023-12-22

**Authors:** Runzhi Guo, Xiang Yao Lam, Liwen Zhang, Weiran Li, Yifan Lin

**Affiliations:** Department of Orthodontics, Peking University School and Hospital of Stomatology, Beijing, China; Division of Paediatric Dentistry and Orthodontics, Faculty of Dentistry, the University of Hong Kong, Hong Kong SAR, China; Department of Dental Medical Center, China-Japan Friendship Hospital, Beijing, China; Department of Orthodontics, Peking University School and Hospital of Stomatology, Beijing, China; Division of Paediatric Dentistry and Orthodontics, Faculty of Dentistry, the University of Hong Kong, Hong Kong SAR, China

**Keywords:** maxillary molar distalization, clear aligner, miniscrew, finite element analysis

## Abstract

**Background/Objectives:**

To compare the biomechanical characteristics of maxillary molar distalization with clear aligners in conjunction with three types of miniscrew anchorage.

**Materials/Methods:**

Three-dimensional (3D) finite element models of maxillary molar distalization with clear aligners and three types of miniscrew anchorage were established, including (A) control group, (B) direct buccal miniscrew anchorage group, (C) direct palatal miniscrew anchorage group, and (D) indirect buccal miniscrew anchorage group. The 3D displacement of maxillary teeth and the principal stress (maximum tensile and compressive stress) on the root and periodontal ligament (PDL) during molar distalization were recorded.

**Results:**

The tooth displacement pattern during maxillary molar distalization in the four groups showed similarities, including labial tipping of anterior teeth, mesial and buccal tipping of premolars, and distal and buccal tipping of molars, but with varying magnitudes. Group C exhibited the greatest molar distalization, with the first molar achieving 0.1334 mm of crown distalization. Group D demonstrated a notable buccal crown movement (0.0682 mm) and intrusion (0.0316 mm) of the first premolar. Compared to Groups A and B, Groups C and D showed less labial crown tipping of the central incisor. Group B showed the greatest amount of maxillary incisor intrusion (central incisor: 0.0145 mm, lateral incisor: 0.0094 mm). Moreover, Groups C and D displayed significantly lower levels of compressive and tensile stress in the roots and PDL of the maxillary central and lateral incisors.

**Limitation:**

Molar distalization is a dynamic process involving sequential tooth movement stages; however, our research primarily examined the tooth movement patterns in the initial aligner.

**Conclusions/Implications:**

The use of miniscrew anchorage, especially direct palatal miniscrew anchorage, may enhance the treatment efficacy of maxillary molar distalization with clear aligners, leading to increased molar distalization, reduced mesial movement of premolars, and minimized labial tipping of anterior teeth.

## Introduction

Clear aligners (CA) have gained popularity as an alternative to traditional braces for orthodontic treatment due to their esthetic appeal, comfort, and convenience [1]. At present, CA is widely used to treat a variety of malocclusions. For patients with mild to moderate Class II malocclusion, maxillary molar distalization could correct the distal molar relationship without the need for premolar extraction. Several studies have indicated maxillary molar distalization as one of the most predictable teeth movements achieved through CA therapies [2–4]. Simon *et al*. reported a high efficacy of 88% in achieving maxillary molar bodily movement of more than 1.5 mm during distalization using CA [5]. Additionally, Auladell *et al*. reported that 3 mm maxillary molar distalization was clinically achievable using CA [6]. However, these studies overlooked the potential anchorage loss, including mesial movement of maxillary premolars and labial movement of maxillary anterior teeth. Besides, the mesial movement of distalized maxillary molar can occur due to reciprocal forces during the retraction of anterior teeth. Considering these factors, the actual efficacy of maxillary molar distalization using CA might not be as high as previously reported [7]. Our recent study found the final efficacy of maxillary molar distalization after CA treatment was only 36.48% after treatment complete [8]. Therefore, effective anchorage control is crucial when performing maxillary molar distalization with CA.

The use of miniscrew is a common practice to reinforce anchorage during orthodontic treatment. During maxillary molar distalization with CA, miniscrews increase the amount of molar distalization and prevent unwanted teeth movements, such as labial movement of anterior teeth [9]. Miniscrews can be categorized as direct or indirect anchorage, depending on the active or passive force delivery, and as palatal or buccal based on their positions. Traditionally, miniscrews are placed buccally as a form of direct anchorage. During distalization, a removable elastic is instructed to be worn from the buccal miniscrew to the precision cut at the maxillary canine. However, a recent study by Cheng *et al*. highlighted that indirect anchorage with buccal miniscrew could significantly increase the efficacy of maxillary molar distalization without relying on patient compliance [10]. Considering another perspective, the placement of palatal miniscrew is generally preferred due to the denser and thicker palatal cortical bone, which offers great stability compared to the buccal cortical bone [11]. Moreover, the interradicular space on the palatal side is typically wider than on the buccal side [12, 13], thus reducing the risk of root damage. However, the utilization of palatal miniscrews in maxillary molar distalization with CA is rarely reported. Jia *et al*. suggested that combining a palatal miniscrew with a patient-specific premolar attachment can reinforce the anchorage [14]. Despite these advancements, the efficacy and biomechanics of maxillary molar distalization using different types of miniscrew anchorage remain unclear.

Finite element analysis (FEA) is a numerical mathematical technique to simulate and analyze the response of teeth subjected to orthodontic force [15]. In the field of orthodontics, FEA has been widely employed to gain a deeper understanding of tooth movement and force application. Several studies have utilized FEA to analyze the biomechanical characteristics of maxillary molar distalization using CA, such as comparisons of different attachments and traction devices [16–18]. While miniscrews play a crucial role in anchorage control during molar distalization, there is a lack of FEA studies investigating the biomechanics of maxillary molar distalization using CA in conjunction with miniscrews. The aim of this FEA study is to compare the biomechanical characteristics in maxillary molar distalization with CA in conjunction with three types of miniscrew anchorage.

## Materials and methods

### Establishment of a three-dimensional (3D) finite element model

A cone beam computed topography (CBCT) scan of a 21-year-old female with Class II division 1 malocclusion was used for the model construction. The patient is appropriate for CA treatment with miniscrew-assisted molar distalization. Specifically, the patient presented an increased overjet of 5 mm, a symmetrical maxillary arch with no crowding, mild maxillary dentoalveolar protrusion (U1-SN = 110.7°), normal crown-root ratio, and sufficient bone at the retromolar area.

First, a total of 528 CBCT images was used to construct a 3D maxillary base model, consisting of maxillary dentition, alveolar bone, and periodontal ligaments (PDL) using MIMICS 20.0 (Materialise, Leuven, Belgium), and further imported into GEOMAGIC Studio 2014 software (Raindrop GEOMAGIC, North Carolina, USA). According to previous studies, the PDL with a uniform thickness of 0.25 mm was fabricated on the outer surface of teeth root, and the thickness of cortical bone was set as 2 mm [19]. The NX1911 software (Siemens, German) was further used to construct CA, attachments and miniscrews. Specifically, the vertical attachments were constructed as follows: 3 mm in height, 2 mm in width, and 1 mm in thickness. The miniscrews were 1.4 mm in diameter and 8 mm in length [14]. Subsequently, the CA was constructed with a 0.75 mm offset extended from the teeth and attachments. Finally, a 3D finite element model was constructed with all components using ANSYS Workbench 2019 (Ansys, Pennsylvania, USA). The right side of maxillary dentition was modeled assuming that the maxillary dentition was symmetric.

### Material properties

The materials used in this study were assumed to be linearly elastic, isotropic and homogenous. As shown in Table 1, the material properties were consistent with previous studies [20]. The 3D finite element models were meshed using ten-node and tetrahedral elements, with the number of nodes and elements for each component outlined in Table 1. The boundary conditions were established by constraining the connection between the root and PDL, PDL and alveolar bone, crown and attachment, and miniscrew and alveolar bone as bonded contact [21]. In addition, a friction contact was established between the CA and teeth and attachment, with a Coulomb frictional coefficient of 0.2 [16].

**Table 1.** Material properties, nodes, and elements.

	Young’ s modulus (MPa)	Poisson rate	Nodes	Elements
Teeth	2 × 10^5^	0.30	51986	83810
Periodontal ligament	0.143	0.45	51526	104245
Cortical bone	1.37 × 10^4^	0.30	12384	24542
Trabecular bone	1370	0.30	14305	35147
Clear aligner	528	0.36	28069	52626
Attachment	1.25 × 10^4^	0.36	22328	34762
Mini-screw	1.14 × 10^5^	0.35	17883	27692

### Establishment of coordinate system

**Figure 1.**
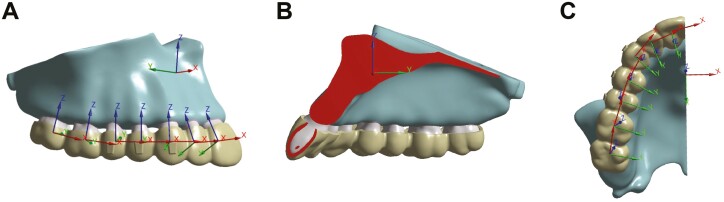
The illustration of coordinate systems of 3D maxillary teeth movement. (A) buccal view; (B) palatal view; (C) occlusal view.

Two coordinate systems were established in this study, as depicted in Fig. 1. The global coordinate system was established to analyze the 3D teeth displacement of the maxillary dentition, with the X-, Y-, and Z-axis representing mesial-distal, buccal-palatal, and vertical directions, respectively. On the other hand, the local coordinate system was established on each maxillary tooth to analyze its individual displacement. For each tooth, the X-axis represented the mesial-distal direction, with a positive value indicating mesial movement and a negative value indicating distal movement. The Y-axis represented the buccal-palatal direction, with a positive value indicating buccal movement and a negative value indicating palatal movement. The Z-axis represented the vertical direction, with a positive value indicating extrusion and a negative value indicating intrusion.

To better understand the movement type, the crown and root of each tooth was analyzed separately. Regarding the crown, specific landmarks were selected, including the midpoint of the incisal edge of the incisors, the cusp of the canine, the buccal cusp of the premolars, and the mesiobuccal cusp of the molars. Regarding the root, the root apex of the anterior teeth, the apex of the buccal root of the first premolar, the root apex of the second premolar, and the apex of the mesiobuccal root of the molars, were selected.

### Experimental design

**Figure 2.**
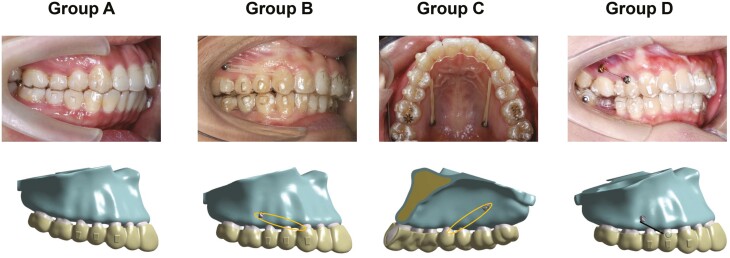
The 3D finite element models and clinical illustration for maxillary molar distalization with CA in conjunction with different types of miniscrew anchorage. (A) control group; (B) direct buccal miniscrew anchorage group; (C) direct palatal miniscrew anchorage group; (D) indirect buccal miniscrew anchorage group.

To stimulate the clinical scenarios, a 0.2-mm distalization was designed for the maxillary first and second molars, resulting in the deformation of CA fitting on the maxillary dentition [16]. Vertical attachments were added to maxillary premolars and canine to counteract the opposing force generated by molar distalization, in accordance with clinical practice. Four models (Fig. 2) were constructed based on the miniscrew position. Details of four models are as below: (A) Control group: no miniscrew was used. (B) Direct buccal miniscrew group: the miniscrew was placed on the buccal alveolar bone, 6 mm away from the alveolar crest, between the roots of the maxillary second premolar and first molar. A 200 g of traction force was applied from the miniscrew to the labial precision cut on the maxillary canine region of the CA [14]. (C) Direct palatal miniscrew group: the miniscrew was placed on the palatal alveolar bone, 8 mm away from the alveolar crest, between the roots of the maxillary first molar and second molar. A 200 g of traction force was applied from the miniscrew to the palatal precision cut on the maxillary first premolar region of the CA. (D) Indirect buccal miniscrew group: the miniscrew was placed on the buccal alveolar bone, 6 mm away from the alveolar crest, between the roots of the maxillary second premolar and first molar. The miniscrew was rigidly connected to the button on the buccal surface of the maxillary first premolar.

### Outcomes

The three-dimensional teeth displacement of the maxillary dentition and individual maxillary teeth during molar distalization were recorded using FEA. The stress on the root and PDL was measured in terms of principal stress, including maximum tensile stress and maximum compressive stress. The principal stress and deformation of CA were also assessed.

## Results

### Three-dimensional analysis of teeth displacement

**Figure 3.**
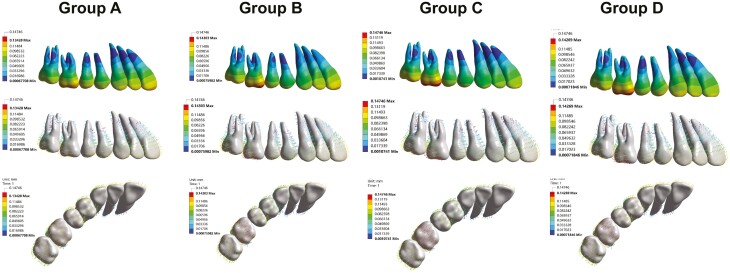
The vector and contour diagrams reflecting the displacement direction and magnitude of maxillary dentition during molar distalization with CA in conjunction with different types of miniscrew anchorage.

Figure 3 shows the overall displacement of all maxillary teeth in the four groups. The maxillary teeth in all groups exhibited a similar displacement pattern, characterized by labial movement of anterior teeth, mesial tipping of premolars, and distal tipping of molars, but with different magnitude of displacement. To gain a better understanding of the specific displacement differences among the groups, the 3D displacement of each maxillary tooth during maxillary molar distalization was analyzed.

Our FEA results revealed that during molar distalization, the maxillary molars exhibited distal and buccal tipping movements in all groups. When compared with the group A, all groups with miniscrews (Groups B, C, and D) exhibited increased amounts of molar distalization. Particularly, Group C showed the greatest distal crown displacement, with the first molar and second molar achieving 0.1334 mm and 0.1164 mm distalization, respectively. Group D displayed a more pronounced buccal tipping of the maxillary first molar, with a buccal crown displacement of 0.0101 mm and a palatal root displacement of 0.0105 mm. Regarding the vertical displacement, a slight extrusion of the maxillary molars was observed in all groups during the distalization process, with the first molar being more affected.

**Figure 4.**
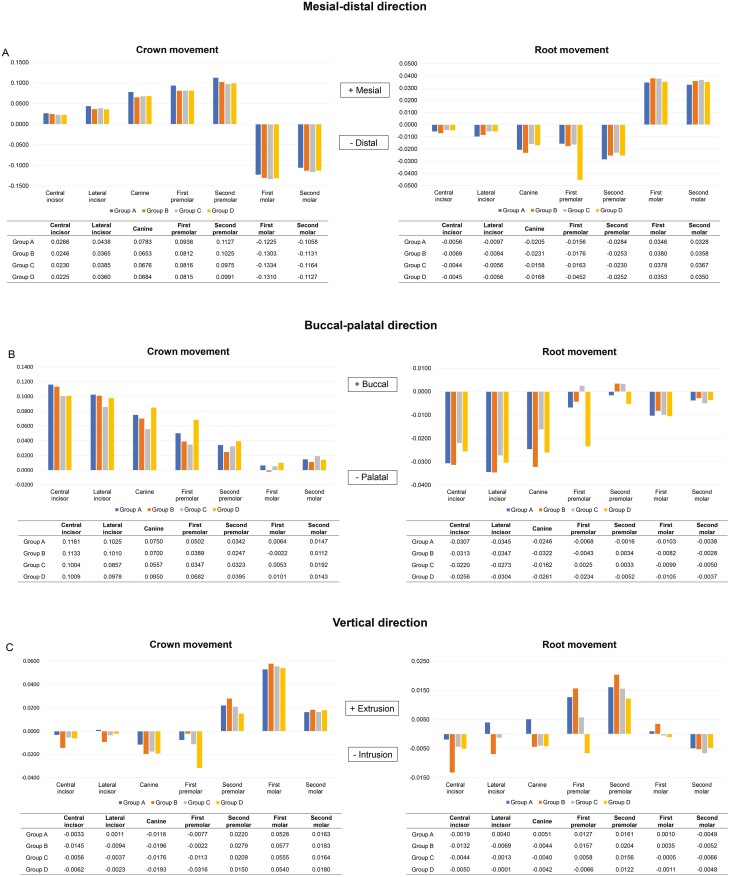
The 3D movement of maxillary teeth in the four groups. (A) mesial-distal direction; (B) buccal-palatal direction; (C) vertical direction.

As shown in Fig. 4, the maxillary premolars in all groups experienced mesial and buccal tipping movement during the molar distalization process. Additionally, the second premolar exhibited extrusion, while the first premolar underwent intrusion. The mesial crown displacement of the second premolar was 0.1127 mm in Group A, 0.1025 mm in Group B, 0.0975 mm in Group C, and 0.0991 mm in Group D, indicating that the use of miniscrews can mitigate the extent of premolar mesial tipping (Fig. 4A). Notably, compared with other groups, Group D demonstrated a substantial buccal crown movement (0.0682 mm) and intrusion (0.0316 mm) of the first premolar.

Across all groups, the maxillary anterior teeth displayed labial movements during molar distalization (Fig. 4B). The central incisor exhibited the most pronounced flaring, following by the lateral incisor and the canine. Compared with the Groups A and B, Groups C and D exhibited less labial crown displacement of the central incisor. The labial crown displacement measured 0.1161 mm in Group A, 0.1133 mm in Group B, 0.1004 mm in Group C, and 0.1009 mm in Group D, respectively. These results suggested that palatal miniscrew and buccal indirect miniscrew could provide better anterior anchorage protection compared to buccal direct miniscrew. Regarding the vertical displacement of the maxillary anterior teeth (Fig. 4C), all miniscrew groups exhibited an intrusive effect. Notably, Group B experienced the greatest amount of anterior teeth intrusion among the four groups, with 0.0145 mm, 0.0094 mm, and 0.0196 mm intrusion observed for the central incisor, lateral incisor, and canine, respectively.

### Stress distribution analysis

To gain insight into the biomechanics of teeth movement and assess the potential risk of root resorption, the principal stress (maximum tensile stress and maximum compressive stress) of roots and PDL of all maxillary teeth during the molar distalization were analyzed.

The principal stress on the root is shown in Table 2. During molar distalization, the miniscrew groups exhibited higher levels of maximum compressive stress in the first and second molars compared to the control group (Supplementary Fig. 1). Among the miniscrew groups, Group C demonstrated the highest tensile and compressive stress levels in the first and second molars (first molar: 0.2200 MPa and 0.2716 MPa; second molar 0.4677 MPa and 0.4297 MPa, respectively), as well as the lowest tensile and compressive stress levels in the second premolar (0.0688 MPa and 0.0531 MPa). In contrast, Group B demonstrated the lowest levels of tensile and compressive stress in the first premolar roots (0.0928 MPa and 0.0750 MPa), while Group D exhibited the highest levels of stress in the first premolar roots (0.2921 MPa and 0.3884 MPa). Regarding maxillary anterior teeth, Groups C and D showed lower levels of maximum compressive stress in the roots of the maxillary central incisor and lateral incisor (Group C: 0.0349 MPa and 0.0628 MPa; Group D: 0.0381 MPa and 0.0696 MPa) compared to the Groups A and B (Group A: 0.0530 MPa and 0.0930 MPa; Group B: 0.0665 MPa and 0.0933 MPa). These results revealed a lower risk of root resorption in the maxillary incisors in Groups C and D. Remarkably, Group C also exhibited lower levels of compressive and tensile stress in the root of the maxillary canine (0.0555 MPa and 0.0705 MPa).

**Table 2.** Maximum tensile stress and maximum compressive stress on root and periodontal ligament (MPa).

	Central incisor		Lateral incisor		Canine		First premolar		Second premolar		First molar		Second molar	
	Tensile	Compressive	Tensile	Compressive	Tensile	Compressive	Tensile	Compressive	Tensile	Compressive	Tensile	Compressive	Tensile	Compressive
Root														
Group A	0.0545	0.0530	0.1176	0.0930	0.1192	0.1249	0.1159	0.1107	0.0923	0.0813	0.1815	0.2193	0.3592	0.3317
Group B	0.0522	0.0665	0.1031	0.0933	0.1131	0.1371	0.0928	0.0750	0.0848	0.0669	0.2013	0.2425	0.4363	0.3993
Group C	0.0349	0.0349	0.0758	0.0628	0.0555	0.0705	0.1185	0.1016	0.0688	0.0531	0.2200	0.2716	0.4677	0.4297
Group D	0.0369	0.0381	0.0962	0.0696	0.1017	0.1184	0.2921	0.3884	0.0824	0.0675	0.2018	0.2465	0.4157	0.3795
Periodontal ligament														
Group A	0.0137	0.0150	0.0146	0.0155	0.0199	0.0184	0.0198	0.0155	0.0380	0.0294	0.0433	0.0335	0.0431	0.0399
Group B	0.0120	0.0164	0.0122	0.0142	0.0129	0.0130	0.0165	0.0097	0.0340	0.0254	0.0500	0.0392	0.0504	0.0516
Group C	0.0108	0.0128	0.0103	0.0118	0.0108	0.0124	0.0099	0.0097	0.0268	0.0201	0.0535	0.0436	0.0546	0.0553
Group D	0.0088	0.0105	0.0123	0.0132	0.0188	0.0206	0.0202	0.0222	0.0308	0.0249	0.0490	0.0388	0.0492	0.0495

Regarding the stress on the PDL (Table 2), Group C exhibited higher maximum compressive stress values on the PDL of the maxillary first molar and second molar compared to the other groups (Group A: 0.0335 MPa and 0.0399 MPa; Group B: 0.0392 MPa and 0.0516 MPa; Group C: 0.0436 MPa and 0.0553 MPa; Group D: 0.0388 MPa and 0.0495 MPa). Consistent with the root stress, Group D displayed a significantly higher level of maximum compressive stress on the PDL of the maxillary canine (0.0206 MPa) and first premolar (0.0222 MPa). In terms of maxillary incisors, the maximum compressive stress on the PDL was concentrated at the labial surface of root (Supplementary Fig. 2). Groups C and D showed significantly lower levels of compressive stress and tensile stress (Group C: 0.0128 MPa and 0.0108 MPa; Group D: 0.0105 MPa and 0.0088 MPa). However, the level of maximum compressive stress in PDL of central incisor was higher in Group B (0.0164 MPa) than that in Group A (0.0150 MPa).

### Stress and deformation analysis of CA

**Figure 5.**
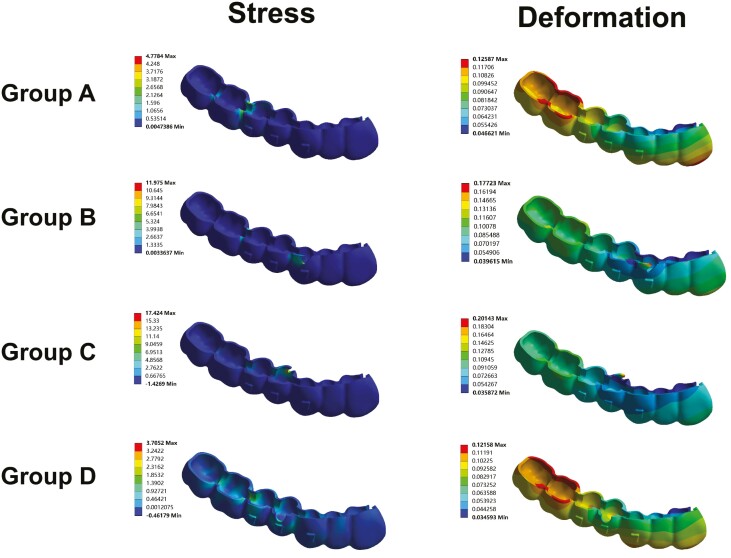
The stress and deformation of CA in the four groups.

The principal stress and deformation of CA were shown in Fig. 5. The maximum stress of CA in direct miniscrew groups (Group B: 11.975 MPa; Group C: 17.424 MPa) was higher than that in the control group (Group A: 4.7784 MPa) and the indirect miniscrew group (Group D: 3.7052 MPa). Similarly, the maximum deformation of CA in the direct miniscrew groups (Group B: 0.17723 mm; Group C: 0.20143 mm) was greater than that in the control group (Group A: 0.12587 mm) and the indirect miniscrew group (Group D: 0.12158 mm). Notably, Group C exhibited the highest maximum stress and deformation among groups. In direct miniscrew groups (Groups B and C), the maximum stress and deformation were located at the precision cut region, whereas in Groups A and D, they were observed at the margins of CA at molars.

## Discussion

Due to the inherent limitations of the material property of CA, the issue of efficacy of maxillary molar distalization with CA should not be overlooked. In clinical practice, Class II elastics and miniscrews are commonly used to enhance treatment efficacy and avoid anchorage loss during molar distalization. However, considering potential risks to temporomandibular joint associated with Class II elastics and the unpredictable nature of patients’ compliance, the use of miniscrews as skeletal anchorage is an efficient approach to maximize desired tooth movement while minimizing undesired tooth movement. Numerous studies have demonstrated the CA in conjunction with miniscrews can achieve optimal treatment outcomes [22–24]. Currently, there are several miniscrew anchorage options available for maxillary molar distalization with CA therapy. Different miniscrew anchorage present with varied biomechanical characteristics, leading to distinct dentoalveolar changes. In this study, we employed FEA to simulate, analyze, and compare the biomechanical effects of molar distalization with CA and three commonly used types of miniscrew anchorage.

In our study, it was found that the utilization of miniscrews, particularly in the palatal miniscrew group, resulted in increased distal displacement of the maxillary molars. The elastic retraction force applied through the aligners indirectly influenced the distal movement of the molars. However, it is important to note that miniscrews alone could not achieve bodily movement of molars; instead, they primarily increased the degree of distal tipping. The distally tipped maxillary molars were not stable and had a tendency to tip mesially after the distalization process. Such instability was confirmed by our previous study, which reported a final efficacy of only 36.48% for maxillary molar distalization following CA treatment [8]. The stability of maxillary molar distalization remains a challenging issue in current CA treatment. To facilitate bodily movement of molars, additional measures such as molar root distal tipping with attachments should be considered.

Undesired effects, including buccal tipping and extrusion movement of maxillary molars, were observed during molar distalization across all groups. Our findings indicated that the buccal tipping was more pronounced in the maxillary second molar compared to the first molar. This phenomenon was particularly evident in the indirect buccal miniscrew group and less pronounced in the direct buccal miniscrew group. The buccal tipping of posterior teeth could potentially result in dental arch expansion. This finding is consistent with our previous study showing that the maxillary dental arch after distalization was significantly wider than the predicted measurements [8]. Regarding vertical changes, the extrusion was more significant in the first molar than the second molar. Among the three miniscrew groups, the extrusion movement of maxillary molars was most notable in the direct buccal miniscrew group. Considering all these factors, the utilization of direct palatal miniscrews may be more favorable for minimizing both buccal tipping and extrusion of the maxillary molars.

The anchorage loss, including mesial movement of premolars and labial movement of maxillary anterior teeth, should not be ignored during the molar distalization. The labial movement of anterior teeth during molar distalization may increase the periodontal risk, such as alveolar bone dehiscence and root resorption, and decrease the treatment efficacy due to reciprocal movement. Our FEA results confirmed the labial flaring of the anterior teeth during molar distalization, and the use of miniscrew could partly reduce this undesirable effect, which is consistent with the findings of Kawamura *et al*. [25]. Despite the utilization of miniscrew anchorage, some degree of anchorage loss still occurred [26]. This can be attributed to the initial contraction force exerted by CA, which exceeded the elastic force of 200 g. As teeth movement progressed, the contraction force of CA decreased while the constant elastic force became dominant, thereby providing additional protection to the anchorage. In the direct miniscrew groups, it is worth noting that the elastic force may cause higher stress and deformation in precision cut region, thereby increasing the risk of CA breakage.

Among three miniscrew groups, the direct palatal miniscrew group demonstrated the least amount of labial tipping in the maxillary anterior teeth, followed by the indirect buccal miniscrew and the direct buccal miniscrew groups. This may be attributed to the fact that the direction of the traction force on the palatal side is close to the center of resistance of the six maxillary anterior teeth. Similarly, the vertical position of anterior teeth also remained stable in direct palatal miniscrew group during molar distalization. In contrast, the buccal direct traction force passed above the center of resistance of the six maxillary anterior teeth, resulting in a labial tipping and intrusion of the maxillary anterior teeth. This conclusion is consistent with the findings reported by Lee *et al*., who similarly demonstrated that palatal miniscrews exhibited superior vertical anchorage control in the maxillary incisors when compared to buccal miniscrews [27].

The stress experienced by root and PDL is closely related to teeth movement and positively correlated with root resorption [28]. The obstruction of PDL blood vessels under heavy forces can increase the risk of root resorption, especially at the apical regions [29]. Among all the maxillary teeth, incisors are more susceptible to root resorption during orthodontic treatment. Therefore, the principal stress of root and PDL were analyzed in this study. The results revealed that the maximum compressive stress on the root and PDL of incisors was lowest in the palatal miniscrew group, and highest in the direct buccal miniscrew group. This can be attributed to the fact in the direct buccal miniscrew group, the retraction force was directly applied to the anterior teeth in close proximity to the root. In contrast, the retraction force was applied to the first premolar in palatal miniscrew group. Besides, the maximum compressive stress on the PDL and root was primarily located at the middle of the labial surface of the root than the apical area. These findings may suggest that palatal miniscrews are an optimal choice for mitigating root resorption in the anterior teeth, as they exert a more favorable distribution of stress on the root and PDL compared to buccal miniscrews.

Regarding maxillary premolars, a mesial and buccal movement was observed in all the groups. The use of miniscrew anchorage proved to be an effective approach in counteracting the mesial force during molar distalization, thereby reducing the mesial tipping of the premolars. Notably, palatal miniscrew anchorage exhibited the least amount of mesial displacement in the second premolar, indicating its effectiveness in anchorage protection. In contrast, the premolars in the indirect buccal miniscrew group exhibited a significant buccal movement, likely attributed to the ligation between the miniscrew and the maxillary first premolar. Vertically, all groups exhibited extrusion of the molars and second premolar, as well as intrusion of the first premolar and anterior teeth, which may exacerbate the maxillary curve of Spee.

In summary, the palatal miniscrew demonstrates favorable biomechanical effects during maxillary molar distalization, including increased molar distalization and reduced anchorage loss, leading to enhanced treatment efficacy. However, it is essential to note that current results only reflect the biomechanical difference of the first aligner among different groups, and such difference may be further magnified or diminished upon using a series of aligners. In addition, the utilization of palatal miniscrew anchorage offers several benefits. The wider palatal interradicular space and denser, thicker palatal cortical bone contribute to increased stability and safety. Unlike buccal miniscrews, which may need repositioning when performing maxillary dentition distalization exceeding 3 mm due to the narrow buccal interradicular space, the palatal miniscrew placed between the maxillary first and second molars does not interfere the distalization process and eliminates the requirement for repositioning.

FEA is a well-established and validated tool for biomechanical analysis of tooth movement during aligner treatment [30, 31]. However, it has some inherent limitations. First, our study only presents the initial teeth displacement, which may not actually represent the their movement with long-term force application of CA and miniscrews. Second, our FEA model assumed a uniform thickness of the PDL, whereas in reality, the PDL has an hourglass shape with the narrowest layer located in the mid-root zone [32]. Third, other factors, such as patient’s age, muscle force, periodontal health, occlusal force and compliance to treatment, can influence teeth movement but were not considered in this study. Therefore, this preliminary FEA study only reflects the biomechanics of maxillary molar distalization in a 21-year-old female patient receiving CA treatment. Besides, the findings of the current FEA require further validation. Further clinical studies are essential to confirm the results of the FEA study.

## Conclusions

Despite the limitations, the use of miniscrew anchorage effectively enhanced the treatment efficacy of maxillary molar distalization with CA. Notably, the CA in conjunction with palatal miniscrew exhibited superior biomechanical characteristics, including increased molar distalization, reduced mesial movement of premolars, minimized labial tipping of anterior teeth, and a lower risk of root resorption.

## Supplementary Material

cjad077_suppl_Supplementary_Figure_S1

cjad077_suppl_Supplementary_Figure_S2

## Data Availability

The datasets used and/or analyzed during the current study are available from the corresponding author on reasonable request.
