# Supplementary figures and images for: Biomechanical analysis of miniscrew-assisted molar distalization with clear aligners: a three-dimensional finite element study
Source: Eur J Orthod. 2023 Dec 22;46(1):cjad077. doi: 10.1093/ejo/cjad077 (PMC10783155; doi:10.1093/ejo/cjad077)

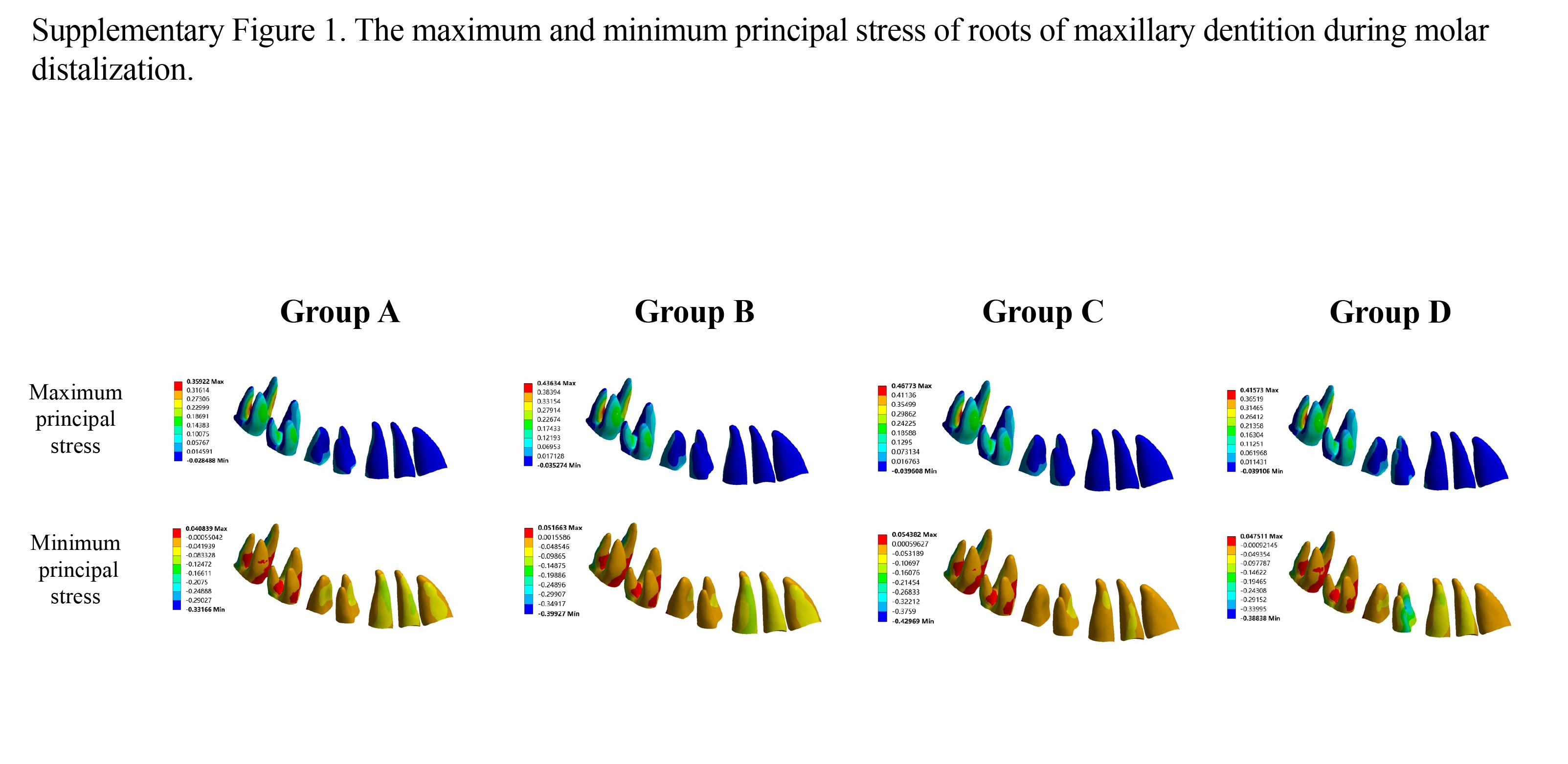

Supplement: cjad077_suppl_Supplementary_Figure_S1 [file cjad077_suppl_supplementary_figure_s1.jpeg]

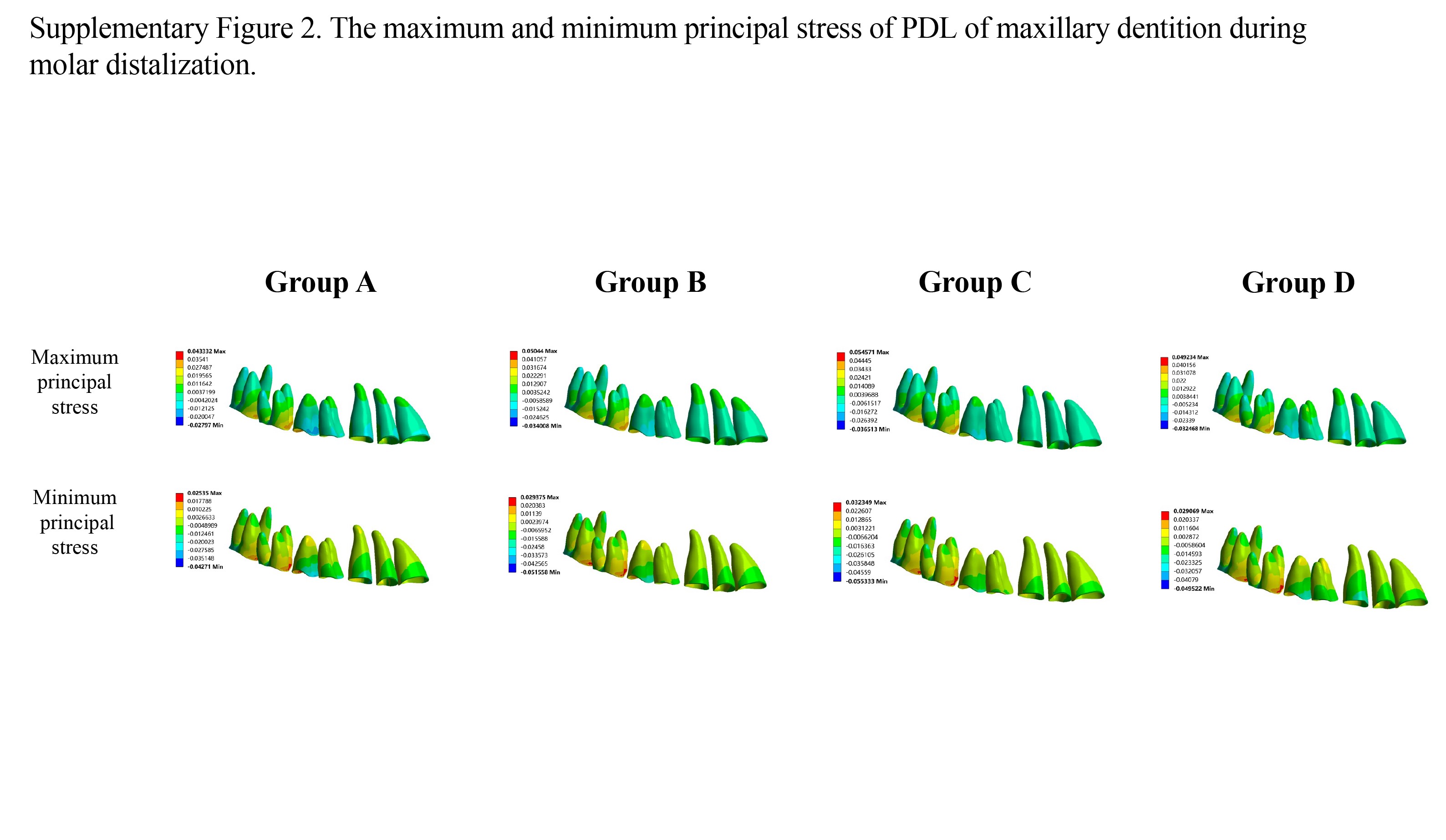

Supplement: cjad077_suppl_Supplementary_Figure_S2 [file cjad077_suppl_supplementary_figure_s2.jpeg]
